# Gender Dimorphism in Skeletal Muscle Leptin Receptors, Serum Leptin and Insulin Sensitivity

**DOI:** 10.1371/journal.pone.0003466

**Published:** 2008-10-21

**Authors:** Borja Guerra, Teresa Fuentes, Safira Delgado-Guerra, Amelia Guadalupe-Grau, Hugo Olmedillas, Alfredo Santana, Jesus Gustavo Ponce-Gonzalez, Cecilia Dorado, José A. L. Calbet

**Affiliations:** 1 Department of Physical Education, University of Las Palmas de Gran Canaria, Campus Universitario de Tafira s/n, Las Palmas de Gran Canaria, Spain; 2 Genetic Unit, Chilhood Hospital-Materno Infantil de Las Palmas, del Sur s/n, Las Palmas de Gran Canaria, Spain; 3 Research Unit, Hospital de Gran Canaria Dr. Negrín, Bco Ballena s/n, Las Palmas de Gran Canaria, Spain; Universidad Europea de Madrid, Spain

## Abstract

To determine if there is a gender dimorphism in the expression of leptin receptors (OB-R170, OB-R128 and OB-R98) and the protein suppressor of cytokine signaling 3 (SOCS3) in human skeletal muscle, the protein expression of OB-R, perilipin A, SOCS3 and alpha-tubulin was assessed by Western blot in muscle biopsies obtained from the m. vastus lateralis in thirty-four men (age = 27.1±6.8 yr) and thirty-three women (age = 26.7±6.7 yr). Basal serum insulin concentration and HOMA were similar in both genders. Serum leptin concentration was 3.4 times higher in women compared to men (P<0.05) and this difference remained significant after accounting for the differences in percentage of body fat or soluble leptin receptor. OB-R protein was 41% (OB-R170, P<0.05) and 163% (OB-R128, P<0.05) greater in women than men. There was no relationship between OB-R expression and the serum concentrations of leptin or 17β-estradiol. In men, muscle OB-R128 protein was inversely related to serum free testosterone. In women, OB-R98 and OB-R128 were inversely related to total serum testosterone concentration, and OB-R128 to serum free testosterone concentration. SOCS3 protein expression was similar in men and women and was not related to OB-R. In women, there was an inverse relationship between the logarithm of free testosterone and SCOS3 protein content in skeletal muscle (r = −0.46, P<0.05). In summary, there is a gender dimorphism in skeletal muscle leptin receptors expression, which can be partly explained by the influence of testosterone. SOCS3 expression in skeletal muscle is not up-regulated in women, despite very high serum leptin concentrations compared to men. The circulating form of the leptin receptor can not be used as a surrogate measure of the amount of leptin receptors expressed in skeletal muscles.

## Introduction

Leptin is a hormone secreted primarily by adipocytes from the white adipose tissue and by the stomach [1], [2] with pleiotropic effects on appetite, energy expenditure, fat deposition, hematopoiesis, angiogenesis, blood pressure, immune function, blood clotting, bone mass, and reproduction [1]. In lean, but not in obese human skeletal muscle, leptin is able to stimulate fatty acid oxidation [3], suggesting that triglyceride accumulation and lipotoxicity in obesity could be caused by changes in the leptin signaling cascade.

There are at least six isoforms of leptin receptors (OB-Rs) generated by mRNA alternative splicing and/or proteolytic processing of the subsequent protein products [4]. These isoforms are divisible into three classes: secreted, short and long. The secreted isoform, also named soluble leptin receptor (sOB-R), is mostly secreted into the bloodstream by the liver [5]. The sOB-R binds circulating leptin and regulates the concentration of free leptin [6]. The short and long isoforms contain identical extracellular and transmembrane domains and differ in the length of the intracellular amino acid sequence [1], [7]. The long form of the leptin receptor (OB-Rb) has a ∼300 residues intracellular domain, highly conserved in several species, and is critical for the effects of this hormone [7]. In fact, the *db*/*db* mice lacking OB-Rb, are phenotypically similar to the leptin-deficient *ob*/*ob* mice and to the *db^3j^*/*db^3j^* mice (which are deficient in all leptin receptor isoforms) [8].

Expression of OB-R mRNA have also been found in non-neuronal tissues [9] such as bone, heart, liver, lung, adrenal glands, testes, spleen, small intestine, pancreatic islets, placenta, adipose tissue and skeletal muscle [10]–[15]. We have recently shown the presence of OB-R protein in human skeletal muscle, adipose tissue and hypothalamus [16].

The concentration of leptin in plasma is proportional to the size of the fat mass but for a given amount of fat mass (and BMI), women have a higher concentration of circulating free leptin [17], [18], [19], i.e. women may be more resistant to the effects of leptin. High leptin levels could down-regulate leptin receptors, since expression (mRNA) of the long (OB-Rb) and short (OB-Ra) isoforms of the leptin receptor are markedly reduced in the hypothalamus and liver of obese rats, which have enhanced plasma leptin concentration [20]. OB-R expression appears to be reduced by testosterone in Leydig cells [21], while estradiol administration to ovariectomized rats increases OB-R protein expression in skeletal muscles [22]. Leptin may also down-regulate leptin signaling in the target tissues by inducing the protein suppressor of cytokine signaling 3 (SOCS3), which blunts JAK-2-dependent leptin signaling [23] and causes leptin resistance in the skeletal muscle [24].

We hypothesized that the high level of circulating leptin observed in women may result in down-regulation of leptin receptors in skeletal muscle or increased SOCS3 protein levels. In addition, we also hypothesized that leptin receptors expression in skeletal muscle will be inversely related to testosterone concentration and directly related to estradiol concentration in both genders. Accordingly, our main purpose was to determine if there is a gender dimorphism in leptin receptor expression in human skeletal muscles. A second purpose was to assess if such dimorphism (if present) is associated with some gender-related factors such as, circulating levels of leptin, testosterone or estradiol concentrations. A third purpose was to determine if the high leptin levels of women are associated to increased SOCS3 protein levels in skeletal muscles. Finally, we aimed at determining if soluble leptin receptor may serve as a surrogate measure of the leptin receptor protein expression in skeletal muscles.

## Materials and Methods

### Materials

The Complete protease inhibitor cocktail was obtained from Roche Diagnostics (Mannheim, Germany). The polyclonal rabbit anti-human leptin receptor antibody that recognizes the human leptin receptor was obtained from Linco Research (St. Charles, Missouri, USA). The polyclonal rabbit anti-human SOCS3 antibody was obtained from Santa Cruz Biotechnology (Santa Cruz, CA, USA). The monoclonal mouse anti-alpha-tubulin antibody was obtained from Biosigma (Madrid, Spain). The secondary HRP-conjugated goat anti-rabbit and donkey anti-mouse antibodies were from Jackson ImmunoReseach (West Grove, PA, USA). The Hybond-P transfer membranes, Hyperfilm ECL and the ECL plus Western Blotting Detection System were from Amersham Biosciences (Little Chalfont, Buckinghamshire, UK). The ChemiDoc XRS System and the image analysis software Quantity One© were obtained from Bio-Rad Laboratories (Hemel Hempstead Hertfordshire, UK).

### Participants

Thirty three healthy men and thirty three healthy women agree to participate in this investigation. The study population was composed by physical education students and police officers. Their levels of physical activity span from an almost sedentary life style to a high level of physical activity. All of them were non-smokers, had normal basal blood glucose concentrations, and had no hypertension or any metabolic disease. The Body composition, basal serum glucose and endocrine variables are shown in Table 1. Written informed consent was obtained from each subject after they received a full explanation about the study procedures. The study was performed in accordance with the Helsinki Declaration of 1975, as revised in 2000, being approved by the Ethical Committee of the University of Las Palmas de Gran Canaria.

**Table 1:** Body composition, basal plasma glucose and endocrine variables.

	Men (n = 34)			Women (n = 33)		
	Mean		SD	Mean		SD
Age (years)	27	±	7	27	±	7
Height (cm)	176.5	±	5.8 *	165.3	±	6.3
Body mass (kg)	76.2	±	11.5 *	60.2	±	8.4
BMI (kg.m^−2^)	24.5	±	3.7 *	22.0	±	2.3
% body fat	18.4	±	7.4 *	28.1	±	7.1
Lean body mass (kg)	58.6	±	5.4 *	40.6	±	3.5
Fat mass (kg)	14.7	±	7.9 *	17.4	±	6.7
Trunk fat mass (kg)	6.9	±	4.8 *	7.0	±	4.5
% fat in trunk	42.8	±	9.4 *	37.6	±	9.0
Glucose (mmol.L^−1^)	4.6	±	0.4 *	4.4	±	0.5
Insulin (pmol.L^−1^)	59.3	±	58.3	53.4	±	24.9
HOMA	1.8	±	2.0	1.5	±	0.7
Leptin (ng.mL^−1^)	4.5	±	4.0 *	15.3	±	8.2
Soluble leptin receptor (ng.mL^−1^)	25.5	±	7.8 *	30.7	±	10.0
Total testosterone (ng.mL^−1^)	7.5	±	3.8 *	1.0	±	0.4
Free testosterone (pg.mL^−1^)	18.0	±	5.7 *	3.7	±	2.1
17β-estradiol	16.0	±	14.4 *	76.9	±	71.0 a

* P<0.05 compared to women. ^a^ (n = 28).

### General Procedures

The body composition of each subject was determined by DXA (Hologic QDR-1500, Hologic Corp., software version 7.10, Waltham, MA) as described elsewhere [25], [26]. On a different day, subjects reported to the laboratory at 8.00 after an overnight fast. After 10 min rest in the supine position a 20 ml blood sample was withdrawn and used to measure serum glucose, insulin, leptin, free and total testosterone, 17β-estradiol, and the soluble leptin receptor. Then a muscle biopsy was obtained from the middle portion of the vastus lateralis muscle using the Bergstrom's technique with suction, as described elsewhere [27]. The muscle specimen was cleaned to remove any visible blood fat or connective tissue. The muscle tissue was immediately frozen in liquid nitrogen, and stored at −80°C for later analysis. In 5 men and five women a small piece of subcutaneous adipose tissue was also sampled 2–3 cm apart from the incision using the same kind of needle.

### Total protein extraction, electrophoresis and Western blot Analysis

For total protein extraction from skeletal muscle and subcutaneous adipose tissue a piece of frozen tissue was homogenized as described elsewhere [16]. Muscle and fat homogenates were rotated end over end at 4°C for 60 min, after which they were centrifuged for 15 min at 20,000 *g* to remove tissue debris. The supernatants were harvested and transferred to clean tubes. An aliquot of each extract was preserved for protein quantification by bicinchoninic acid assay. Proteins were solubilized in sample buffer containing 0.0625 mM Tris-HCl, pH 6.8, 2.3% [w/v] SDS, 10% [v/v] glycerol, 5% [v/v] β-mercaptoethanol, 0.001% [w/v] bromophenol blue. Equal amounts (50 µg) of each sample were electrophoresed on 7.5–10% sodium dodecyl sulfate – polyacrylamide gel electrophoresis (SDS-PAGE) using the system of Laemmli [28] and transferred to Hybond-P membranes according to the method of Towbin et al. [29].

For immunoblotting, membranes were pre-incubated with 5% blotting grade blocker non-fat dry milk (Bio-Rad Laboratories, Hercules, CA, USA) in Tris-buffered saline (TBS) with 0.1% Tween 20 (blotto blocking buffer) for 1 h at room temperature (20–22°C). To detect the leptin receptor isoforms (OB-Rs), membranes were incubated with a rabbit polyclonal specific anti-human OB-R (long form) antibody.

To detect SOCS3 protein expression membranes were incubated with a rabbit polyclonal specific anti-human SOCS3 antibody. To control for differences in loading and transfer efficiency across membranes, an antibody directed against alpha-tubulin was used to hybridize on the same samples. Membrane incubations with polyclonal rabbit anti-OB-R (diluted 1∶2.000 in blotto blocking buffer) and with polyclonal rabbit anti-SOCS3 (diluted 1∶500 in blotto blocking buffer) were performed over night at 4°C. Membrane incubations with monoclonal mouse anti-alpha-tubulin (diluted 1∶70,000 in blotto blocking buffer) were performed for 1 h at room temperature.

As control for adipose tissue protein presence in muscular tissue, a polyclonal rabbit anti-perilipin A antibody was used [30]. To explore the expression of this protein in human skeletal muscle and subcutaneous adipose tissue, membranes were blocked with 4% Bovine Serum Albumin (Sigma, Madrid, Spain) in TBS with 0.1% Tween 20 (BSA blocking buffer) for 1 h at room temperature. Membrane incubations with polyclonal rabbit anti-perilipin A antibody (diluted 1∶1,500 in BSA blocking buffer) were performed for 1 h at room temperature. Antibody-specific labeling was revealed by incubation with a HRP-conjugated goat anti-rabbit antibody (1∶20,000) or a HRP-conjugated donkey anti-mouse (1∶10,000) antibody both diluted in blotto blocking buffer and visualized with the ECL chemiluminescence kit (Amersham Biosciences). Specific bands were visualized with the ECL chemiluminiscence kit, visualized with the ChemiDoc XRS system (Bio-Rad Laboratories) and analyzed with the image analysis program Quantity one© (Bio-Rad laboratories). Data are reported as band intensity of immunostaining values (arbitrary units) obtained for OB-R, Perilipin or SOCS3 relative to those obtained for alpha-tubulin. Alpha-tubulin content in the male and female muscle biopsies was similar (6.54±0.44 and 5.44±0.42 arbitrary units of immunostaining band density, respectively, *P*>0.05).

### Glucose and insulin measurements

Serum glucose was measured by the hexokinase method using Gluco-quant reagents (Roche/Hitachi, 11876899216, Indianapolis, USA). Serum insulin was measured by an electrochemiluminiscence immunoassay (ECLIA) intended for use on Modular Analytics analyzer E170 using Insulin kit reagents (Roche/Hitachi, Indianapolis, USA). In a first incubation, insulin from 20 µl serum sample, a biotinylated monoclonal insulin-specific antibody and a monoclonal insulin-specific antibody labeled with a ruthenium complex form a sandwich complex. After addition of streptavidin-coated microparticles, the complex becomes bound to the solid phase via interaction of biotin and streptavidin. The reaction mixture is aspirated into the measuring cell where the microparticles are magnetically captured onto the surface of the electrode. Unbound substances are then removed by washing. Application of a voltage to the electrode then induces chemiluminescent emission which is measured by a photomultiplier. Results are determined via a calibration curve. Insulin sensitivity was 0.20 µU/ml.

### Assessment of insulin resistance

In each subject, the degree of insulin resistance was estimated at the baseline by the Homeostasis model assessment (HOMA) according to the method described by Matthews et al. [31].

### Leptin Assays

Serum leptin were determined by Enzyme-Linked Immunosorbent Assay (ELISA) (ELx800 Universal Microplate Reader, Bioteck Instruments Inc, Vermont, USA), using reagent kits from Linco Research (#EZHL-80SK, Linco ResearchSt. Charles, Missouri, USA) and following the manufacturer's instructions. The sensitivity of the total leptin assays was 0.05 ng/mL. The intra-assay coefficient variation was 3.8% and the inter-assay coefficient of variation was 4.4%.

### Soluble leptin receptor (sOB-R) Assays

Serum OB-Rs were determined by Enzyme-Linked Immunosorbent Assay (ELISA) (ELx800 Universal Microplate Reader, Bioteck Instruments Inc, Vermont, USA), using reagent kits from R&D Systems (#DOBR00, R&D, Minneapolis, MN, USA) and following the manufacturer's instructions. The sensitivity of the sOB-R assays was 0.057 ng/mL. The intra-assay coefficient variation was 4.4% and the inter-assay coefficient of variation was 6.8%.

### Total and Free Testosterone Assays

Serum free and total testosterone were determined by Enzyme-Linked Immunosorbent Assay (ELISA) (ELx800 Universal Microplate Reader, Bioteck Instruments Inc, Vermont, USA), using reagent kits from IBL (#DB52181 for free testosterone and #RE52151 for total testosterone, IBL, Hamburg, Germany) and following the manufacturer's instructions. The sensitivity of the free testosterone and total testosterone assays was 0.17 pg/mL and 0.08 ng/mL, respectively. The intra-assay coefficient variation was 6.1% and 3.6%, for free and total testosterone respectively. The inter-assay coefficient of variation for free and total testosterone was 7.8% and 7.1%, respectively.

### 17β-Estradiol Assay

17β-estradiol was measured by a competitive electrochemiluminiscence immunoassay (ECLIA) intended for use on Modular Analytics analyzer E170 using E2 reagents (Roche/Hitachi, 03000079122, Indianapolis, USA). Briefly, by incubating 35 µl of serum sample with an estradiol-specific biotinylated antibody, an immunocomplex is formed, the amount of which is dependent upon the analyte concentration in the sample. After addition of streptavidin-coated microparticles and an estradiol derivative labeled with a ruthenium complex, the final antibody-hapten complex was bound to a solid phase via a biotin-streptavidin interaction. After remove the unbound substances, application of a voltage induces chemiluminiscent emission which is measured by a photomultiplier. Results were determined via a calibration curve being the analytical sensitivity 18.4 pmol/L.

### Statistical analysis

Variables were checked for normal distribution by using a Kolmogorov-Smirnov test with the Lilliefors correction. When necessary, the analysis was done on logarithmically transformed data. Gender differences were determined with ANOVA. To determine if there was a gender difference in 128 KDa OBR isoform content in the muscle biopsies we used ANCOVA with perilipin A as a covariate. The relationship between variables was determined using linear regression analysis. Values are reported as the mean±standard deviation. P≤0.05 was considered significant. Statistical analysis was performed using SPSS v.8.0 for Windows (SPSS Inc., Chicago, IL).

## Results

Body composition, anthropometrics and hormonal data are reported in Table 1. Both genders were comparable in age, but women were smaller and had lower body mass and higher percentage of body fat compared to men (all, P<0.05). The proportion of fat accumulated in the trunk was greater in men than women (P<0.05).

### Serum leptin concentrations, HOMA and sexual hormones

**Figure 1:**
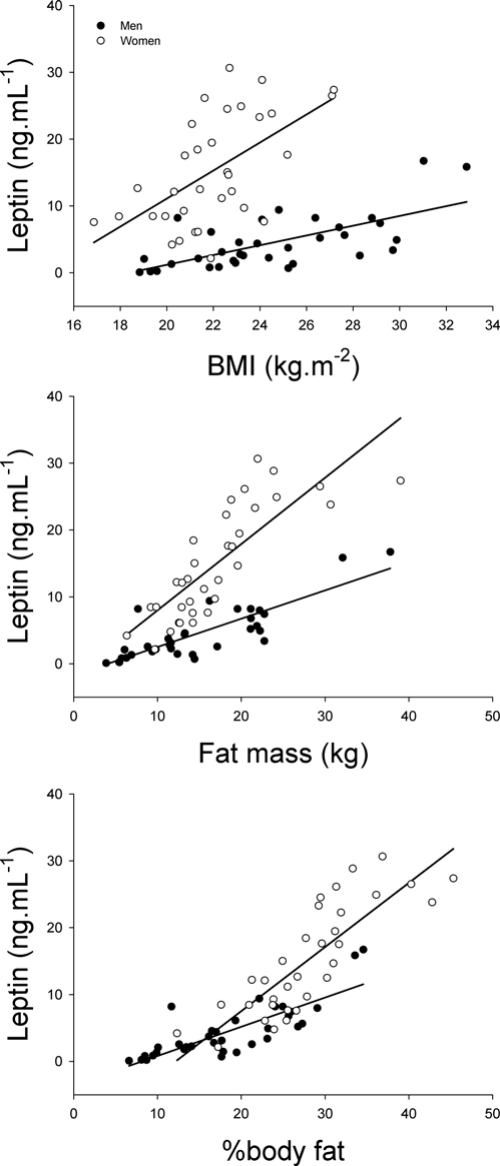
Relationship between the basal leptin concentration in serum and body mass index (BMI), whole body fat mass, and percentage of body fat.

Although basal serum glucose concentration was 5% lower in women than men (P<0.05), basal serum insulin concentration and HOMA were similar in both genders. Serum leptin concentration was 3.4 times higher in women compared to men (P<0.05) and this difference remained significant after accounting for the differences in percentage of body fat. In the whole group, there was a relationship between the percentage of body fat and the serum leptin concentration (r = 0.85, P<0.001), and also in each gender separately (r = 0.81 and r = 0.83, in men and women, respectively, both P<0.001) (Fig. 1).

In men, but not in women, leptin concentration was inversely associated to total serum testosterone concentration (r = −0.38, p<0.05). In men, but not in women, serum total and free testosterone concentration was inversely associated with the percentage of body fat (r = −0.51 and r = −0.41, respectively, both P<0.01). On the other hand, a similar trend was observed between the logarithm of serum leptin concentration and 17β-estradiol in women (r = −0.34, P = 0.07, n = 28).

The logarithm of HOMA was associated to the serum leptin concentration in men (r = 0.64, P<0.001) and women (r = 0.63, P<0.001). In both genders, the logarithm of HOMA was also associated to the percentage of body fat (r = 0.54 and r = 0.59, in men and women, both P<0.001). After accounting for the differences in percentage of body fat the association between the logarithm of HOMA and serum leptin concentration remained significant in men (r = 0.57, P<0.05), but not in women (r = 0.31, P = 0.09). 001).

### Expression of leptin receptors in skeletal muscle shows a gender dimorphism that can not be explained by differences in serum leptin concentrations or fat tissue infiltration

**Figure 2:**
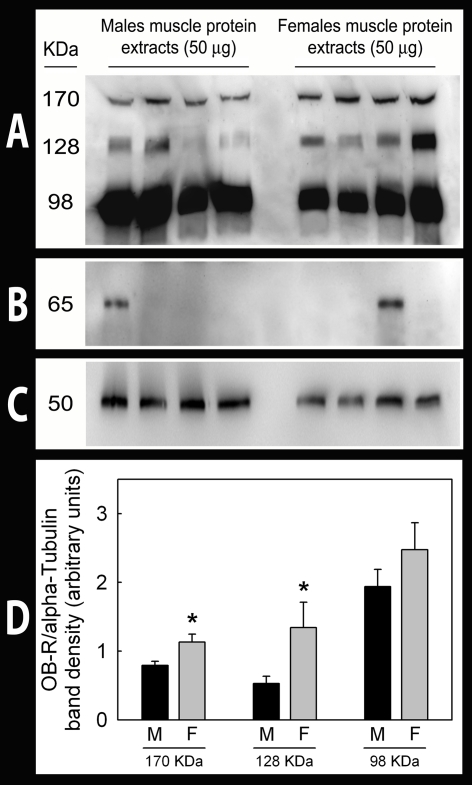
Leptin receptor (OB-R) isoform protein expression in male and female human skeletal muscle. Total Protein extracts were prepared from male and female muscle and OB-R, perilipin A and alpha-tubulin protein expression was analyzed by Western blot. A: a representative immunoblot assay after incubation with a polyclonal rabbit anti-OB-R antibody specifically raised against de long isoform of the leptin receptor. B: a representative western blot after incubation with a polyclonal rabbit anti-perilipin A antibody in the same samples used in A. C: a representative immunoblot analysis after incubation with the monoclonal mouse anti-alpha-tubulin antibody in the same samples used in A. D: densitometric immunosignal values (arbitrary units of band densities) of OB-R bands relative to those obtained for alpha-tubulin.

Leptin receptor protein expression in skeletal muscle was 41% (OB-R170, P<0.05) and 163% (OB-R128, P<0.05) greater in women than men (Fig. 2). No significant between genders differences in OB-R98 expression were observed (OB-R98; P = 0.14). There was no relationship between leptin receptors in skeletal muscle and serum leptin concentration in either group, even after accounting for differences in serum sOB-R concentration. In men, muscle OB-R128 protein was inversely associated to serum free testosterone (r = −0.34, P = 0.05). In women, OB-R98 and OB-R128 were inversely associated to total serum testosterone concentration (r = −0.39 and r = −0.36, respectively, both P<0.05), and OB-R128 to serum free testosterone concentration (r = −0.36, P<0.05). No relationship was observed in men or in women between skeletal muscle OB-R protein content and 17β-estradiol concentration in serum.

Perilipin A content in skeletal muscle extracts was 93% higher in women than in men (P<0.001). However, perilipin A content in the adipose tissue was similar in men (n = 5) and women (n = 5). OB-R98 and perilipin A were associated (r = 0.42 and r = 0.54, in men and women, respectively, both P<0.05). In women, but not in men, perilipin A correlated also with OB-R128 protein in skeletal muscle (r = 0.50, P<0.01). Nevertheless, gender differences in OB-R128 expression remained after accounting for differences in perilipin A content.

### The soluble leptin receptor can not be used a surrogate measure of leptin receptors protein in skeletal muscle

The sOB-R was 20% higher in women than men (P<0.05) and in men correlated with serum free testosterone concentration (r = 0.35, P<0.05) (Table 1). There was no relationship between sOB-R concentration in serum and OB-R isoforms in skeletal muscle. There was no relationship between HOMA and muscle leptin receptors or sOB-R.

### SOCS3 protein content in skeletal muscle is similar in healthy men and women and is not related to serum leptin concentration

**Figure 3:**
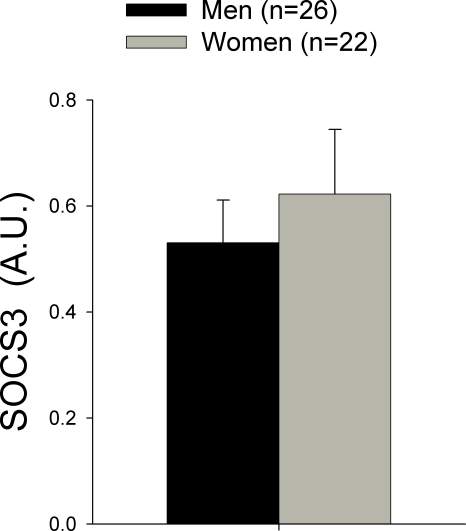
Suppressor of cytokine signaling 3 (SOCS3) protein content in muscle biopsies obtained from the musculus vastus lateralis in men and women. A.U.: arbitrary units.

**Figure 4:**
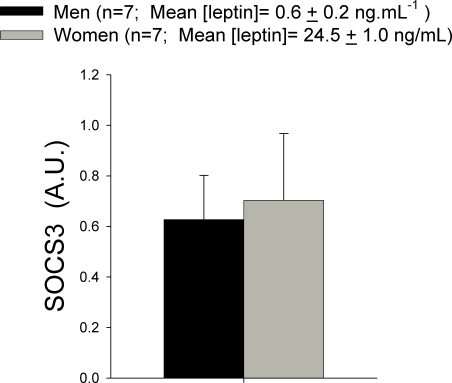
Suppressor of cytokine signaling 3 (SOCS3) protein content in muscle biopsies obtained from the musculus vastus lateralis in men the seven men with the lowest serum leptin concentrations and the seven women with the highest serum leptin concentrations. A.U.: arbitrary units.

SOCS3 protein expression was similar in men and women, despite the fact that women had about 4-folds higher leptin concentration than men (Fig. 3). Even when we took the seven men with the lowest leptin concentration and compared them with the seven women with the highest leptin concentration, SOCS3 expression was comparable in both groups, despite 40-folds higher leptin concentration in women than men (Fig. 4). In women, there was an inverse association between the logarithm of free testosterone and SCOS3 protein content in skeletal muscle (r = −0.46, P<0.05).

## Discussion

In this study we provide further evidence for the presence of the long (Ob-Rb) and short isoforms of leptin receptor protein in human skeletal muscle [16]. Immunoblot analysis detected several immunoreactive proteins with molecular weight of 98, 128 and 170 kDa. The most prominent 128 kDa band has a molecular weight similar to OB-Rb, based on its amino acid composition [32]. The smaller 98 kDa protein is likely to correspond to one of the short leptin receptor isoforms [4]. The 170 kDa has a molecular weight which corresponds well with the glycosylated form of OB-Rb [33].

Based on the experimental evidences showing: 1) that in conditions with chronically elevated leptin concentration, such as obesity and pregnancy the expression of OB-Rb mRNA is reduced in the hypothalamus [34], but also in peripheral tissues such as the liver [34]; 2) that acute leptin administration causes an acute reduction in the expression of leptin receptors in cell lines [34]; 3) that prolonged fasting in humans increases OB-R mRNA in peripheral mononuclear cell [35]; and 4) that administration of human recombinant leptin in fasting humans blunts the increase in OB-R in mononuclear cells [35], we hypothesized that women compared to men will have reduced protein expression of OBR in skeletal muscle. In contrast with our hypothesis, this study shows that the 170 and 128 KDa leptin receptors isoforms are more abundant in female than male skeletal muscle. We have also observed that the muscle biopsies obtained from women had a greater amount of perilipin A than those from men. Inasmuch as perilipin A is a protein present in adipocytes and absent in skeletal muscle fibers, greater perilipin A content in women's skeletal muscle biopsies strongly suggest that women have more intermuscular adipose tissue than men [36]. These findings only apply to healthy young humans and different results may be possible in patients with obesity or metabolic diseases.

Greater intermuscular adipose tissue could explain part of the gender difference in OB-R128 protein content. Nevertheless, after accounting for differences in perilipin content, OB-R128 protein content was still 2.3 times higher in women than men. On the other hand, adipose tissue contamination in the skeletal muscle biopsies does not explain the gender differences in OB-R170, since this isoform was not detected in subcutaneous adipose tissue.

It has been postulated that the sexual dimorphism in leptin levels reflects reduced leptin sensitivity in women [37] however, our findings are more compatible with increased leptin sensitivity in the women's skeletal muscle, unless the intracellular signaling pathways are more inhibited in women than men.

### Regulation of leptin receptor expression

In agreement with previous studies an inverse association was observed between leptin concentration and serum testosterone in men [38], likely caused by an inhibitory effect of leptin on Leydig cells steroidogenesis [39] and perhaps in testosterone biosynthesis [40]. In turn, androgens reduce leptin gene transcription in rat adipocytes [41] and testosterone administration to young men reduces serum leptin [42]. This effect is likely due a direct inhibition of leptin production in adipocytes [43], likely combined an increased leptin clearance rate and shortened plasma leptin half-life [44].

Although animal studies have shown that 17β-estradiol administration to ovariectomized rats increases plasma leptin levels [45] by stimulating leptin production in the adipocytes [41], leptin also inhibits steroidogenesis in granulosa cells of the ovary [46], what could explain our findings in regard with the negative relationship between 17β-estradiol and leptin in women. In humans, leptin changes in the same direction as 17β-estradiol during the menstrual cycle [47], [48]. Ovarian stimulation with human FSH (225 IU daily) during an in vitro fertilization program led to a concomitant rise of plasma leptin coupled to the elevation of plasma 17β-estradiol [47]. However, postmenopausal women have higher plasma leptin levels than weight-matched men [48] and the same as premenopausal women after accounting for differences in fat mass [17]. The latter implies that at the most 17β-estradiol and androgen could only explain a small part of the sexual dimorphism in plasma leptin concentrations [17].

Although no relationship was observed in the present study between 17β-estradiol concentration and skeletal muscles leptin receptors we can not rule out estrogens as contributors to the sexual dimorphism in skeletal muscle leptin receptors in humans, mainly because a punctual isolated determination of basal plasma concentration of 17β-estradiol give just a rough estimation of the estrogenic action on the muscles at mid and long term, particularly fertile women [47], [48]. In fact, a recent study has shown that in ovariectomized rats 17β-estradiol increases OB-Rb protein in skeletal muscle [22]. Nevertheless our findings indicate that small differences in 17β-estradiol concentration do not account for individual differences in muscle leptin receptors in women or men.

### Increased skeletal muscle leptin receptors in women

The sOB-R, a circulating soluble form of the leptin receptor is the main leptin binding protein in blood and determines the free fraction of circulating leptin [35], [49], [50]. Administration of leptin to humans has been reported to elicit small reciprocal changes in sOB-R plasma concentration [35]. The latter agrees with our observation of slightly lower serum soluble receptor leptin concentration in women than men.

Our study indicates that female skeletal muscle has the potential to respond more to leptin stimulation due to the remarkably greater abundance of leptin receptors, particularly of the two main isoforms involved in intracellular leptin signaling (OB-Rb and OB-Ra) [51]. This could explain why women have an increased capacity to oxidize fatty acids during prolonged exercise than men [52], [53].

It remains unknown which is the mechanism that determines this sexual dimorphism in skeletal muscle leptin receptors. Our study indicates that leptin itself does not explain the sexual dimorphism in skeletal muscle OB-R expression, since despite a broad spectrum of leptin concentration is both genders, there was no correlation between serum leptin concentration and the abundance of leptin receptors in skeletal muscle, even after accounting for the differences in serum sOB-R concentration. Nevertheless, our study provides indirect evidence supporting a role of serum free testosterone concentration which could explain 12–13% of the variability in skeletal muscle content of the 128 KDa leptin receptor isoform in both genders.

Although leptin and insulin share some intracellular signaling pathways, our study indicates that insulin (at basal concentration) does not appear to play a role in the regulation of leptin receptor expression in skeletal muscles. In agreement, Liu et al. 2007 [20] reported no significant relationship between OB-Ra or OB-Rb gene expression in the hypothalamus and liver and serum insulin concentrations in obese rats.

### Soluble leptin receptor does not reflect the amount of leptin receptors in skeletal muscle

The sOB-R could originate from alternative splicing of the leptin receptor or from full-length functional leptin receptors released by enzymatic cleavage, most likely in the liver [5]. In the latter case, it has been hypothesized that serum sOB-R concentration could reflect the amount of leptin receptor expressed in tissues [54]. Our study, however, shows that sOB-R is not related to the expression of OB-R isoforms in skeletal muscles.

### SOCS3 protein content in skeletal muscles

It has been reported that SOCS-3 mRNA levels are increased in the skeletal muscle of type 2 diabetic patients compared with control subjects and correlates with reduced insulin-stimulated glucose uptake [55]. Skeletal muscle SOCS-3 mRNA is also increased in obese mice [56]. The study of SOCS3 mRNA levels in subcutaneous adipose tissue of humans has yielded contradicting results. It has been reported to be increased in a mixed sample (9 men and 7 women) [55] and to be reduced in obese women [57]. Thus, it has been suggested that this contradicting results could have been cause by gender differences in the expression of SOCS3 in the obese human [57]. Here we report for the first time SOCS3 protein levels in human skeletal muscle in both genders. In contrast with our hypothesis, SOCS3 protein is not up-regulated in women compared to men, implying that if women have some degree of leptin resistance in their skeletal muscles the mechanisms is not related to SOCS3 up-regulation.

In summary, this study shows that in healthy young humans there is a sexual dimorphism in the expression of leptin receptors in human skeletal muscles, which can not be explained by differences in circulating leptin or soluble leptin receptor, or differences in intermuscular adipose tissue. A small part of the sexual dimorphism could be explained by an inverse relationship between serum free testosterone concentration and the 128 KDa isoform of the leptin receptor. No relationship was observed between estradiol concentration and leptin receptors in skeletal muscle. The circulating form of the leptin receptor can not be used as a surrogate measure of the amount of leptin receptors expressed in skeletal muscles. Despite the fact that the female skeletal muscle is exposed to very high leptin concentrations, SOCS3 protein expression was not up-regulated, indicating that if women have some degree of leptin resistance in their skeletal muscles the mechanism should be other than SOCS3 up-regulation.
